# Multiple Extrauterine Adenomyomas Presenting in Upper Abdomen and Pelvis: A Case Report and Brief Review of the Literature

**DOI:** 10.1155/2012/565901

**Published:** 2012-11-27

**Authors:** Mana Moghadamfalahi, Daniel S. Metzinger

**Affiliations:** ^1^Department of Pathology and Laboratory Medicine, University of Louisville Hospital, 530 South Jackson Street, Louisville, KY 40202, USA; ^2^Division of Gynecologic Oncology, James Graham Brown Cancer Center, 529 South Jackson Street, Louisville, KY 40202, USA

## Abstract

Adenomyomas are benign tumors composed of smooth muscle and endometrial tissue. These tumors usually arise from the myometrium. Extrauterine adenomyomas are rare with only a few case reports available in the literature. Here, we report an unusual case of multiple adenomyomas in a 39-year-old woman six years after hysterectomy for multiple leiomyomata. To the best of our knowledge, this is the first case of extrauterine adenomyoma presenting as an upper abdominal mass.

## 1. Introduction

Uterine adenomyomas are benign tumors composed of smooth muscles, endometrial glands, and endometrial stroma. They are distinguished from adenomyosis by their sharp demarcation from the surrounding normal tissues, and from leiomyomas by the presence of intrinsic endometrial glandular and stromal elements [1–3].

An extrauterine location is extremely rare and to the best of our knowledge, only four cases of extrauterine adenomyoma have been reported in the English literature. We are reporting a fifth case, and only the first case in the upper abdomen. The differential diagnosis includes uterine-like mass lesions, parasitic leiomyoma, disseminated peritoneal leiomyomatosis, endomyometriosis, and leiomyoma with entrapped benign endometrial tissue.

In this case study, we will focus on this rare entity with a brief review of the literature, discussion about differential diagnosis, possible pathophysiology, and treatment options.

## 2. Case Report

**Figure 1:**
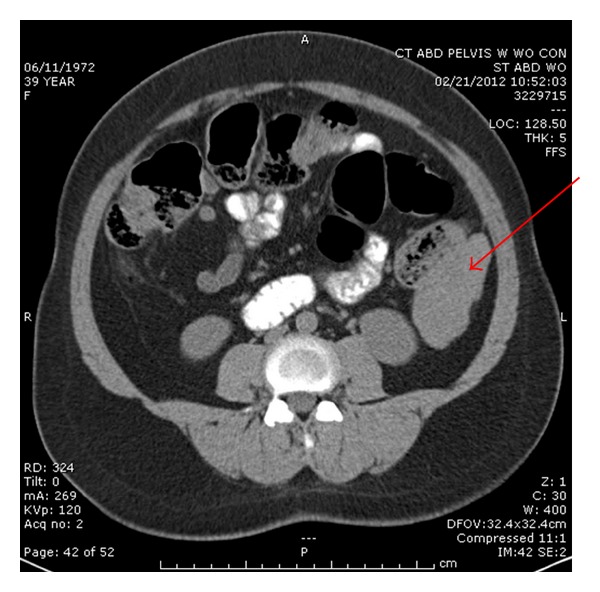
CT scan of the left upper abdominal mass with heterogeneity, adjacent to the descending colon.

The patient was a 39-year-old woman who presented with complaints of abdominal pain and rectal bleeding. A computed tomography (CT) scan of the abdomen and pelvis was performed that showed one mass in her left paracolic gutter (Figure 1) and one mass in the right hemipelvis.

Her medical history was significant for a supracervical morcellated hysterectomy in 2006 for multiple leiomyomata, removal of a cervical leiomyoma a few months ago, and history of endometriosis.

At laparotomy, the patient had an approximately 6.0 centimeter mass in the cul-de-sac. It was free from either ovary or tube and was not attached to the cervix. It was attached to the upper part of the rectum and was below the vagina. There was also a secondary mass in the left upper quadrant of the abdomen which was 7.5 cm. On gross examination, both masses appeared to be encapsulated with a tan-white firm homogenous cut surface and multiple areas of cystic changes with dark brown viscous fluid.

A frozen section was performed and interpreted as leiomyoma with foci of endometriosis most consistent with adenomyoma.

**Figure 2:**
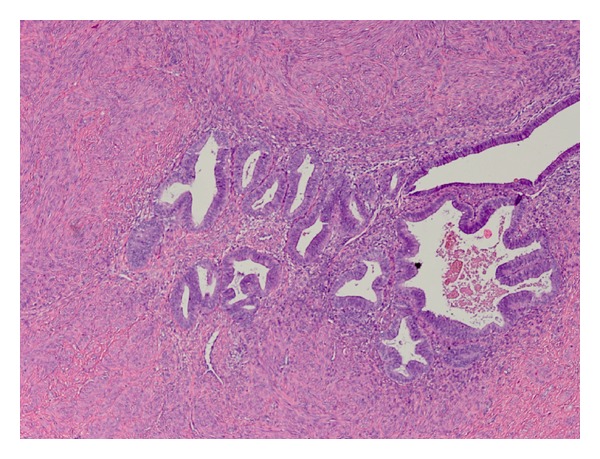
Histologic appearance of the mass with bland smooth muscle fascicles and foci of endometrial like tissue.

**Figure 3:**
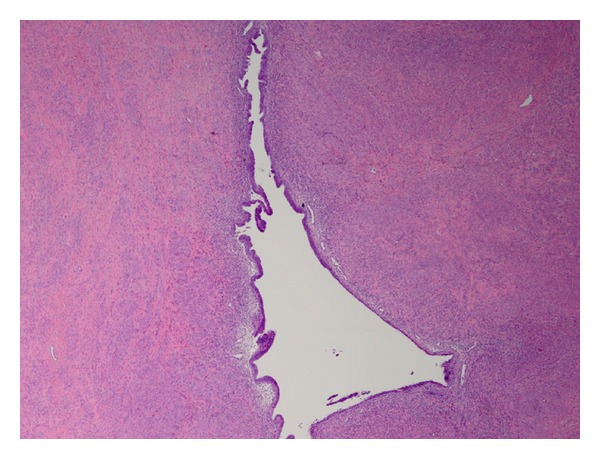
Multiple foci of endometrial like tissue identified throughout the mass.

A microscopic evaluation showed benign anastomosing fascicles of smooth muscle cells. No nuclear atypia or necrosis was noted. Foci of cystic changes lined with endometrial glands and stroma were randomly distributed throughout both masses. No distinct central cavity was identified (Figures 2 and 3).

The microscopic slides from the patient's previous hysterectomy from 2006 were also reviewed and showed multiple leiomyomata. No evidence of adenomyosis or intrauterine adenomyoma was noted.

## 3. Discussion

All of the previously described cases of extrauterine adenomyomas have been confined to the pelvis (in ovarian ligament, in periadnexal or parametrial soft tissue) [3].

In this paper we document a fifth case in this series and only the first one in the upper abdomen.

The mean age in these cases was 51 years (range 39–65 years). Most reported cases presented with a single mass and have ranged in size from 0.8 cm to 6.3 cm.

The differential diagnosis includes uterine-like mass lesions, parasitic leiomyoma, disseminated peritoneal leiomyomatosis, endomyometriosis, and leiomyoma with entrapped benign endometrial tissue.

The uterine-like mass lesions typically show an organoid arrangement consisting of a single central cavity lined by endometrial-type epithelium with surrounding myometrial-like tissue [3]. The lesions described as extrauterine adenomyomas are composed of what appears to be a leiomyoma with scattered foci of endometriosis without making a distinct central cavity.

Several cases of “extrauterine adenomyoma with uterine-like mass features or uterine-like mass with features of extrauterine adenomyoma” have been documented in the literature. Here, we emphasize the differences between the two entities.

It is also important to mention that some of the reported cases of uterine-like mass lesions have been associated with renal or urinary tract anomalies. It is possible that some of the uterine-like mass lesions arise as a result of congenital abnormalities such as müllerian duct fusion defects [2, 3].

Parasitic leiomyoma develops when a uterine leiomyoma or fragments of a leiomyoma detach and implant on the abdominopelvic cavity and gradually grow to become a clinically significant lesion [4, 5]. This phenomenon has been recently described to be more commonly associated with morcellated hysterectomies [5]. In our case, even though the patient underwent a morcellated hysterectomy six years ago, she did not have any evidence of intrauterine adenomyoma or adenomyosis. Therefore, foci of cycling endometrial type tissue in the extrauterine lesions exclude this possibility.

Disseminated peritoneal leiomyomatosis is excluded because of the presence of cycling endometrial tissue, large size of the lesions, and lack of recent or current pregnancy.

The lesions described in this paper were well-circumscribed masses composed of intersecting short fascicles of smooth muscle bundles reminiscent of uterine leiomyoma or adenomyoma, and there was no adhesion or obvious continuity with surrounding normal stromal tissue. This excludes the possibility of endomyometriosis.

Another possible differential diagnosis of benign leiomyoma with entrapped, not intrinsic, endometrial tissue has been suggested. Similar lesions have been described within the uterus [2, 3]. Lack of adhesions, no evidence of intrauterine adenomyoma or adenomyosis and serial sectioning with no evidence of adjacent endometriosis excludes this diagnosis.

So far two theories are proposed to explain the etiology of the extrauterine adenomyoma: (a) the uterine/müllerian duct fusion defect theory and (b) the subcoelomic mesenchyme transformation theory. The first theory explains the abnormality in the development of the female genital tract. Each male or female fetus has two pairs of genital ducts: wolffian (mesonephric) and müllerian (paramesonephric). The müllerian duct, as the main female genital duct, begins as a longitudinal folding of the coelomic epithelium on the anterolateral surface of the urogenital ridge. Lack of fusion of the müllerian duct system may explain various duplications or atresias of the uterus [6]. The same etiology has been proposed to be related to uterine-like mass lesions.

The subcoelomic mesenchyme is a layer of tissue that lies underneath the mesothelial surface of the peritoneum. In fetus, this layer of tissue gives rise to the mesenchyme of the urogenital ridge that surrounds the early müllerian and wolffian ducts. In adults, the subcoelomic mesenchyme presents as an inconspicuous layer of flattened cells that lie immediately underneath the subserosal stroma of the uterus, ovaries, tubes, and uterine ligaments. The cells of this layer, which is also called secondary müllerian system, are thought to be multipotential and can proliferate in response to hormonal stimulation [6].

The extrauterine adenomyomas in our patient are best explained by the second theory of subcoelomic mesenchyme transformation. The patient in our case did not have any evidence of urogenital abnormality, so it is less likely that she had a müllerian duct fusion defect.

Long-term followup of patients with extrauterine adenomyomas has been documented in a study by Carinelli et al. for the first time. They reported two cases of multiple extrauterine adenomyomas and uterine-like masses. They observed a relapse in one of their patients after six months. In both cases, surgery followed by long-term therapy with monthly GnRH agonist to stop the estrogen-stimulated proliferation of the neoplastic tissue prevented recurrence for long periods of observation (4 and 10 years in their experience) [7]. This suggests a treatment option for these patients to prevent possible recurrence and complications from having multiple surgeries.
